# The lived experience of people with obesity: study protocol for a systematic review and synthesis of qualitative studies

**DOI:** 10.1186/s13643-021-01706-5

**Published:** 2021-06-21

**Authors:** Emma Farrell, Marta Bustillo, Carel W. le Roux, Joe Nadglowski, Eva Hollmann, Deirdre McGillicuddy

**Affiliations:** 1 School of Education, University College Dublin, Belfield, Dublin 4, Ireland; 2 University College Dublin Library, Dublin, Ireland; 3 Diabetes Complications Research Centre, University College Dublin, Dublin, Ireland; 4 Obesity Action Coalition, Tampa, USA

**Keywords:** Obesity, Lived experience, Patient experience, Obesity treatment, Qualitative

## Abstract

**Background:**

Obesity is a prevalent, complex, progressive and relapsing chronic disease characterised by abnormal or excessive body fat that impairs health and quality of life. It affects more than 650 million adults worldwide and is associated with a range of health complications. Qualitative research plays a key role in understanding patient experiences and the factors that facilitate or hinder the effectiveness of health interventions. This review aims to systematically locate, assess and synthesise qualitative studies in order to develop a more comprehensive understanding of the lived experience of people with obesity.

**Methods:**

This is a protocol for a qualitative evidence synthesis of the lived experience of people with obesity. A defined search strategy will be employed in conducting a comprehensive literature search of the following databases: PubMed, Embase, PsycInfo, PsycArticles and Dimensions (from 2011 onwards). Qualitative studies focusing on the lived experience of adults with obesity (BMI >30) will be included. Two reviewers will independently screen all citations, abstracts and full-text articles and abstract data. The quality of included studies will be appraised using the critical appraisal skills programme (CASP) criteria. Thematic synthesis will be conducted on all of the included studies. Confidence in the review findings will be assessed using GRADE CERQual.

**Discussion:**

The findings from this synthesis will be used to inform the EU Innovative Medicines Initiative (IMI)-funded SOPHIA (Stratification of Obesity Phenotypes to Optimize Future Obesity Therapy) study. The objective of SOPHIA is to optimise future obesity treatment and stimulate a new narrative, understanding and vocabulary around obesity as a set of complex and chronic diseases. The findings will also be useful to health care providers and policy makers who seek to understand the experience of those with obesity.

**Systematic review registration:**

PROSPERO CRD42020214560.

**Supplementary Information:**

The online version contains supplementary material available at 10.1186/s13643-021-01706-5.

## Background

Obesity is a complex chronic disease in which abnormal or excess body fat (adiposity) impairs health and quality of life, increases the risk of long-term medical complications and reduces lifespan [1]. Operationally defined in epidemiological and population studies as a body mass index (BMI) greater than or equal to 30, obesity affects more than 650 million adults worldwide [2]. Its prevalence has almost tripled between 1975 and 2016, and, globally, there are now more people with obesity than people classified as underweight [2].

Obesity is caused by the complex interplay of multiple genetic, metabolic, behavioural and environmental factors, with the latter thought to be the proximate factor which enabled the substantial rise in the prevalence of obesity in recent decades [3, 4]. This increased prevalence has resulted in obesity becoming a major public health issue with a resulting growth in health care and economic costs [5, 6]. At a population level, health complications from excess body fat increase as BMI increases [7]. At the individual level, health complications occur due to a variety of factors such as distribution of adiposity, environment, genetic, biologic and socioeconomic factors [8]. These health complications include type 2 diabetes [9], gallbladder disease [10] and non-alcoholic fatty liver disease [11]. Excess body fat can also place an individual at increased cardiometabolic and cancer risk [12–14] with an estimated 20% of all cancers attributed to obesity [15].

Although first recognised as a disease by the American Medical Association in 2013 [16], the dominant cultural narrative continues to present obesity as a failure of willpower. People with obesity are positioned as personally responsible for their weight. This, combined with the moralisation of health behaviours and the widespread association between thinness, self-control and success, has resulted in those who fail to live up to this cultural ideal being subject to weight bias, stigma and discrimination [17–19]. Weight bias, stigma and discrimination have been found to contribute, independent of weight or BMI, to increased morbidity or mortality [20].

Thomas et al. [21] highlighted, more than a decade ago, the need to rethink how we approach obesity so as not to perpetuate damaging stereotypes at a societal level. Obesity research then, as now, largely focused on measurable outcomes and quantifiable terms such as body mass index [22, 23]. Qualitative research approaches play a key role in understanding patient experiences, how factors facilitate or hinder the effectiveness of interventions and how the processes of interventions are perceived and implemented by users [24]. Studies adopting qualitative approaches have been shown to deliver a greater depth of understanding of complex and socially mediated diseases such as obesity [25]. In spite of an increasing recognition of the integral role of patient experience in health research [25, 26], the voices of patients remain largely underrepresented in obesity research [27, 28].

Systematic reviews and syntheses of qualitative studies are recognised as a useful contribution to evidence and policy development [29]. To the best of the authors’ knowledge, this will be the first systematic review and synthesis of qualitative studies focusing on the lived experience of people with obesity. While systematic reviews have been carried out on patient experiences of treatments such as behavioural management [30] and bariatric surgery [31], this review and synthesis will be the first to focus on the experience of living with obesity rather than patient experiences of particular treatments or interventions. This focus represents a growing awareness that ‘patients have a specific expertise and knowledge derived from lived experience’ and that understanding lived experience can help ‘make healthcare both effective and more efficient’ [32].

This paper outlines a protocol for the systematic review of qualitative studies based on the lived experience of people with obesity. The findings of this review will be synthesised in order to develop an overview of the lived experience of patients with obesity. It will look, in particular, at patient concerns around the risks of obesity and their aspirations for response to obesity treatment.

## Methods

The review protocol has been registered within the PROSPERO database (registration number: CRD42020214560) and is being reported in accordance with the reporting guidance provided in the Preferred Reporting Items for Systematic Reviews and Meta-Analyses Protocols (PRISMA-P) statement [33, 34] (see checklist in Additional file 1).

### Information sources and search strategy

The primary source of literature will be a structured search of the following electronic databases (from January 2011 onwards—to encompass the increase in research focused on patient experience observed over the last 10 years): PubMed, Embase, PsycInfo, PsycArticles and Dimensions. There is no methodological agreement as to how many search terms or databases out to be searched as part of a ‘good’ qualitative synthesis (Toye et al. [35]). However, the breadth and depth of the search terms, the inclusion of clinical and personal language and the variety within the selected databases, which cover areas such as medicine, nursing, psychology and sociology, will position this qualitative synthesis as comprehensive. Grey literature will not be included in this study as its purpose is to conduct a comprehensive review of peer-reviewed primary research. The study’s patient advisory board will be consulted at each stage of the review process, and content experts and authors who are prolific in the field will be contacted. The literature searches will be designed and conducted by the review team which includes an experienced university librarian (MB) following the methodological guidance of chapter two of the JBI Manual for Evidence Synthesis [36]. The search will include a broad range of terms and keywords related to obesity and qualitative research. A full draft search strategy for PubMed is provided in Additional file 2.

### Eligibility criteria

Studies based on primary data generated with adults with obesity (operationally defined as BMI >30) and focusing on their lived experience will be eligible for inclusion in this synthesis (Table 1). The context can include any country and all three levels of care provision (primary, secondary and tertiary). Only peer-reviewed, English language, articles will be included. Studies adopting a qualitative design, such as phenomenology, grounded theory or ethnography, and employing qualitative methods of data collection and analysis, such as interviews, focus groups, life histories and thematic analysis, will be included. Publications with a specific focus, for example, patient’s experience of bariatric surgery, will be included, as well as studies adopting a more general view of the experience of obesity.

**Table 1:** Inclusion and exclusion criteria based on modified PICoS (Methley et al.,[37])

PICoS	Inclusion criteria	Exclusion criteria
Population	People with experience of obesity (BMI >30) Adults (18 years and over)	People without experience of obesity (BMI >30) Children (under 18 years)
Phenomenon of interest	Patient’s lived experience	Experiences and opinions of professionals working with people with obesity
Context	Any country Primary, secondary and tertiary care	
Study type	Qualitative Focused on patient experience Original research Mixed methods	Quantitative

### Screening and study selection process

Search results will be imported to Endnote X9, and duplicate entries will be removed. Covidence [38] will be used to screen references with two reviewers (EF and EH) removing entries that are clearly unrelated to the research question. Titles and abstracts will then be independently screened by two reviewers (EF and EH) according to the inclusion criteria (Table 1). Any disagreements will be resolved through a third reviewer (DMcG). This layer of screening will determine which publications will be eligible for independent full-text review by two reviewers (EF and EH) with disagreements again being resolved by a third reviewer (DMcG).

### Data extraction

Data will be extracted independently by two researchers (EF and EH) and combined in table format using the following headings: author, year, title, country, research aims, participant characteristics, method of data collection, method of data analysis, author conclusions and qualitative themes. In the case of insufficient or unclear information in a potentially eligible article, the authors will be contacted by email to obtain or confirm data, and a timeframe of 3 weeks to reply will be offered before article exclusion.

### Quality appraisal of included studies

This qualitative synthesis will facilitate the development of a conceptual understanding of obesity and will be used to inform the development of policy and practice. As such, it is important that the studies included are themselves of suitable quality. The methodological quality of all included studies will be assessed using the critical appraisal skills programme (CASP) checklist, and studies that are deemed of insufficient quality will be excluded. The CASP checklist for qualitative research comprises ten questions that cover three main issues: Are the results of the study under review valid? What are the results? Will the results help locally? Two reviewers (EF and EH) will independently evaluate each study using the checklist with a third and fourth reviewer (DMcG and MB) available for consultation in the event of disagreement.

### Data synthesis

The data generated through the systematic review outlined above will be synthesised using thematic synthesis as described by Thomas and Harden [39]. Thematic synthesis enables researchers to stay ‘close’ to the data of primary studies, synthesise them in a transparent way and produce new concepts and hypotheses. This inductive approach is useful for drawing inference based on common themes from studies with different designs and perspectives. Thematic synthesis is made up of a three-step process. Step one consists of line by line coding of the findings of primary studies. The second step involves organising these ‘free codes’ into related areas to construct ‘descriptive’ themes. In step three, the descriptive themes that emerged will be iteratively examined and compared to ‘go beyond’ the descriptive themes and the content of the initial studies. This step will generate analytical themes that will provide new insights related to the topic under review.

Data will be coded using NVivo 12. In order to increase the confirmability of the analysis, studies will be reviewed independently by two reviewers (EF and EH) following the three-step process outlined above. This process will be overseen by a third reviewer (DMcG). In order to increase the credibility of the findings, an overview of the results will be brought to a panel of patient representatives for discussion. Direct quotations from participants in the primary studies will be italicised and indented to distinguish them from author interpretations.

### Assessment of confidence in the review findings

Confidence in the evidence generated as a result of this qualitative synthesis will be assessed using the Grading of Recommendations Assessment, Development and Evaluation Confidence in Evidence from Reviews of Qualitative Research (GRADE CERQual) [40] approach. Four components contribute to the assessment of confidence in the evidence: methodological limitations, relevance, coherence and adequacy of data. The methodological limitations of included studies will be examined using the CASP tool. Relevance assesses the degree to which the evidence from the primary studies applies to the synthesis question while coherence assesses how well the findings are supported by the primary studies. Adequacy of data assesses how much data supports a finding and how rich this data is. Confidence in the evidence will be independently assessed by two reviewers (EF and EH), graded as high, moderate or low, and discussed collectively amongst the research team.

### Reflexivity

For the purposes of transparency and reflexivity, it will be important to consider the findings of the qualitative synthesis and how these are reached, in the context of researchers’ worldviews and experiences (Larkin et al, 2019). Authors have backgrounds in health science (EF and EH), education (DMcG and EF), nursing (EH), sociology (DMcG), philosophy (EF) and information science (MB). Prior to conducting the qualitative synthesis, the authors will examine and discuss their preconceptions and beliefs surrounding the subject under study and consider the relevance of these preconceptions during each stage of analysis.

### Dissemination of findings

Findings from the qualitative synthesis will be disseminated through publications in peer-reviewed journals, a comprehensive and in-depth project report and presentation at peer-reviewed academic conferences (such as EASO) within the field of obesity research. It is also envisaged that the qualitative synthesis will contribute to the shared value analysis to be undertaken with key stakeholders (including patients, clinicians, payers, policy makers, regulators and industry) within the broader study which seeks to create a new narrative around obesity diagnosis and treatment by foregrounding patient experiences and voice(s). This synthesis will be disseminated to the 29 project partners through oral presentations at management board meetings and at the general assembly. It will also be presented as an educational resource for clinicians to contribute to an improved understanding of patient experience of living with obesity.

## Discussion

Obesity is a complex chronic disease which increases the risk of long-term medical complications and a reduced quality of life. It affects a significant proportion of the world’s population and is a major public health concern. Obesity is the result of a complex interplay of multiple factors including genetic, metabolic, behavioural and environmental factors. In spite of this complexity, obesity is often construed in simple terms as a failure of willpower. People with obesity are subject to weight bias, stigma and discrimination which in themselves result in increased risk of mobility or mortality. Research in the area of obesity has tended towards measurable outcomes and quantitative variables that fail to capture the complexity associated with the experience of obesity. A need to rethink how we approach obesity has been identified—one that represents the voices and experiences of people living with obesity. This paper outlines a protocol for the systematic review of available literature on the lived experience of people with obesity and the synthesis of these findings in order to develop an understanding of patient experiences, their concerns regarding the risks associated with obesity and their aspirations for response to obesity treatment. Its main strengths will be the breadth of its search remit—focusing on the experiences of people with obesity rather than their experience of a particular treatment or intervention. It will also involve people living with obesity and its findings disseminated amongst the 29 international partners SOPHIA research consortium, in peer reviewed journals and at academic conferences. Just as the study’s broad remit is its strength, it is also a potential challenge as it is anticipated that searchers will generate many thousands of results owing to the breadth of the search terms. However, to the best of the authors’ knowledge, this will be the first systematic review and synthesis of its kind, and its findings will contribute to shaping the optimisation of future obesity understanding and treatment.

## Supplementary Information

**Additional file 1:.** PRISMA-P (Preferred Reporting Items for Systematic review and Meta-Analysis Protocols) 2015 checklist: recommended items to address in a systematic review protocol*.

**Additional file 2: Table 1**. Search PubMed search string.

## Data Availability

Not applicable.

## Abbreviations

BMI: Body mass index
CASP: Critical appraisal skills programme
GRADE CERQual: Grading of Recommendations Assessment, Development and Evaluation Confidence in Evidence from Reviews of Qualitative Research
IMI: Innovative Medicines Initiative
MeSH: Medical Subject Headings
PICoS: Population, phenomenon of interest, context, study type
SOPHIA: Stratification of Obesity Phenotypes to Optimize Future Obesity Therapy
