# Supplementary material for: The lived experience of people with obesity: study protocol for a systematic review and synthesis of qualitative studies
Source: Syst Rev. 2021 Jun 21;10:181. doi: 10.1186/s13643-021-01706-5 (PMC8215772; doi:10.1186/s13643-021-01706-5)
Supplement: Supplementary file 1 — Additional file 1:. PRISMA-P (Preferred Reporting Items for Systematic review and Meta-Analysis Protocols) 2015 checklist: recommended items to address in a systematic review protocol*. [file 13643_2021_1706_MOESM1_ESM.doc]

**PRISMA-P (Preferred Reporting Items for Systematic review and Meta-Analysis Protocols) 2015 checklist: recommended items to address in a systematic review protocol***

| Section and topic | Item No | Checklist item |
| --- | --- | --- |
| ADMINISTRATIVE INFORMATION | | |
| Title: |  |  |
| Identification | 1a | Protocol |
| Update | 1b | N/A |
| Registration | 2 | PROSPERO CRD42020214560 |
| Authors: |  |  |
| Contact | 3a | Dr Emma Farrell, School of Education, University College Dublin. [emma.farrell@ucd.ie](mailto:emma.farrell@ucd.ie) (Corresponding Author)  Dr Marta Bustillo, University College Dublin Library. [marta.bustillo@ucd.ie](mailto:marta.bustillo@ucd.ie)  Prof. Carel W le Roux, Diabetes Complications Research Centre, University College Dublin, [carel.leroux@ucd.ie](mailto:carel.leroux@ucd.ie)  Joe Nadglowski, President/CEO Obesity Action Coalition. [jnadglowski@obesityaction.org](mailto:jnadglowski@obesityaction.org)  Eva Hollmann, School of Education, University College Dublin. [eva.hollmann@ucdconnect.ie](mailto:eva.hollmann@ucdconnect.ie)  Dr Deirdre McGillicuddy. School of Education, University College Dublin. mailto:deirdre.mcgilicuddy@ucd.ie |
| Contributions | 3b | EF conceptualised and designed the protocol with input from DMcG and MB. EF drafted the initial manuscript. EF and MB defined the concepts and search items with input from DmcG, CleR and JN. MB and EF designed and executed the search strategy. DMcG, CleR, JN and EH provided critical insights and reviewed and revised the protocol. All authors have approved and contributed to the final written manuscript. |
| Amendments | 4 | Important protocol amendments will be documented and recorded on PROSPERO and reflected in any resulting publications |
| Support: |  |  |
| Sources | 5a | None |
| Sponsor | 5b | This project has received funding from the Innovative Medicines Initiative 2 Joint Undertaking (JU) under grant agreement No 875534. The JU receives support from the European Union’s Horizon 2020 research and innovation programme and EFPIA and T1D Exchange, JDRF, and Obesity Action Coalition. |
| Role of sponsor or funder | 5c | The funding body had no role in the design of the study and will not have a role in collection, analysis, and interpretation of data or in writing the manuscript. |
| INTRODUCTION | | |
| Rationale | 6 | Obesity is a prevalent, complex, progressive and relapsing chronic disease characterised by abnormal or excessive body fat that impairs health and quality of life. It effects more than 650 million adults worldwide and is associated with a range of health complications. Qualitative research plays a key role in understanding patient experiences and the factors that facilitate or hinder the effectiveness of health interventions. This review aims to systematically locate, assess and synthesise qualitative studies in order to develop a more comprehensive understanding of the lived experience of people with obesity. To the best of the authors knowledge, it will be the first systematic review of its kind. |
| Objectives | 7 | What is the qualitative nature of the lived experience of adults (18+) with obesity (BMI 30+). |
| METHODS | | |
| Eligibility criteria | 8 | | Inclusion and exclusion criteria will be based on a modified PICoS. Studies based on primary data generated with adults with obesity (operationally defined as BMI >30) and focusing on their lived experience will be eligible for inclusion in this synthesis. Studies adopting a qualitative design and employing qualitative methods of data collection and analysis, such as interviews, focus groups, life histories and thematic analysis, will be included. Publications with a specific focus, for example patients experience of bariatric surgery, will be included, as will studies adopting a more general view of the experience of obesity.   | PICoS | Inclusion Criteria | Exclusion Criteria | | --- | --- | --- | | Population | People with experience of obesity (BMI >30)  Adults (18 years and over) | People without experience of obesity (BMI >30)  Children (under 18 years) | | Phenomenon of interest | Patient’s lived experience | Experiences and opinions of professionals working with people with obesity | | Context | Any country  Primary, secondary and tertiary care |  | | Study Type | Qualitative  Focused on patient experience  Original research  Mixed methods | Quantitative | | | --- | --- | --- | --- | --- | --- | --- | --- | --- | --- | --- | --- | --- | --- | --- | --- | |
| Information sources | 9 | The following databases will be searched for relevant studies: PubMed, PsycInfo (ProQuest interface), PsycArticles (ProQuest interface), Embase, and Dimensions. Grey literature will not be included in this study as its purpose is to conduct a comprehensive review of peer reviewed primary research. |
| Search strategy | 10 | Sample Search Strategy for PubMed. No limits or search filters will be used.  "Obesity/psychology"[Majr] OR "Obesity, Morbid/psychology"[Mesh] OR "Overweight/psychology"[Majr] OR "Body Mass Index"[Mesh] OR "Hyperphagia/psychology"[Mesh] OR "Food Addiction/psychology"[Mesh] OR obese OR “morbid obesity” OR overweight OR "over weight" OR overeat OR "over eat” OR "body mass index" OR BMI OR hyperphagia OR "food addiction"  AND  "Narration"[Mesh] OR "Narrative Medicine"[Mesh] OR "Patient-Centered Care"[Mesh] OR "Interviews as Topic"[Mesh] OR "Personal Narratives as Topic"[Mesh] OR "Biographies as Topic"[Mesh] OR "Autobiographies as Topic"[Mesh] OR narration OR “narrative medicine” OR “patient-centered care” OR interview OR biography OR autobiography OR “personal narrative*” OR "lived experience" OR "patient narrative*" OR "patient story" |
| Study records: |  |  |
| Data management | 11a | Search result records will be imported into an Endnote X9 library, de-duplicated and subsequently imported into the Covidence systematic review platform for review. |
| Selection process | 11b | Results will be screened initially by a single reviewer (EF) to remove entries that are clearly unrelated to the research question. Titles and abstracts will then be independently screened by two reviewers (EF and EH) according to the inclusion criteria (Table 3). Any disagreements will be resolved through a third reviewer (DMcG). This layer of screening will determine which publications will be eligible for independent full-text review by two reviewers (EF and EH) with disagreements again being resolved by a third reviewer (DMcG). The methodological quality of all included studies will be assessed using the critical appraisal skills programme (CASP) checklist and studies that are deemed of insufficient quality will be excluded. The CASP checklist for qualitative research comprises ten questions that cover three main issues: Are the results of the study under review valid? What are the results? Will the results help locally? Two reviewers (EF and EH) will independently evaluate each study using the checklist with a third and fourth reviewer (DMcG and MB) available for consultation in the event of disagreement. |
| Data collection process | 11c | Data from the findings of selected primary studies will be coded line-by-line using NVivo 12 coding software. In order to increase the confirmability of the analysis, studies will be reviewed independently by two reviewers (EF and EH). This process will be overseen by a third reviewer (DMcG). |
| Data items | 11d | Authors, research aims, participant details, data collection methods, methods of data analysis, findings, author conclusions. There will be no pr-eplanned data assumptions and simplifications. |
| Outcomes and prioritization | 13 | As this will be a systematic review of qualitative data, there will be no pre-definition or prioritisation of outcomes. Once the research meets the PICoS and CASP criteria, the emerging outcomes will be accepted. |
| Risk of bias in individual studies | 14 | In acknowledgement of the difficulties associated with assessing risk of reporting biases (Page, McKenzie & Higgins, 2018), the authors will rely on study level explication of bias as included in the CASP checklist. Studies that do not explicate bias, the relationship between researcher and participants, and/or the process by which data were managed and analayised (amongst other criteria) will not considered to meet the CASP criteria and will therefore be excluded prior to synthesis. |
| Data synthesis | 15a | N/A |
|  | 15b | N/A |
|  | 15c | N/A |
|  | 15d | The data generated through the systematic review will be synthesised using thematic synthesis as described by Thomas and Harden (2008). Thematic synthesis is made up of a three-step process. Step one consists of line by line coding of the findings of primary studies. The second step involves organising these ‘free codes’ into related areas to construct ‘descriptive’ themes. In step three, the descriptive themes that emerged will be iteratively examined and compared to ‘go beyond’ the descriptive themes and the content of the initial studies.  In order to increase the credibility of the findings, an overview of the results will be brought to a panel of patient representatives for discussion. |
| Meta-bias(es) | 16 | See above (14) |
| Confidence in cumulative evidence | 17 | Confidence in the evidence generated as a result of this qualitative synthesis will be assessed using the Grading of Recommendations Assessment, Development and Evaluation Confidence in Evidence from Reviews of Qualitative Research (GRADE CERQual) approach. |

*** It is strongly recommended that this checklist be read in conjunction with the PRISMA-P Explanation and Elaboration (cite when available) for important clarification on the items. Amendments to a review protocol should be tracked and dated. The copyright for PRISMA-P (including checklist) is held by the PRISMA-P Group and is distributed under a Creative Commons Attribution Licence 4.0.**

*From: Shamseer L, Moher D, Clarke M, Ghersi D, Liberati A, Petticrew M, Shekelle P, Stewart L, PRISMA-P Group. Preferred reporting items for systematic review and meta-analysis protocols (PRISMA-P) 2015: elaboration and explanation. BMJ. 2015 Jan 2;349(jan02 1):g7647.*
