# Supplementary material for: The lived experience of people with obesity: study protocol for a systematic review and synthesis of qualitative studies
Source: Syst Rev. 2021 Jun 21;10:181. doi: 10.1186/s13643-021-01706-5 (PMC8215772; doi:10.1186/s13643-021-01706-5)
Supplement: Supplementary file 2 — Additional file 2: Table 1. Search PubMed search string. [file 13643_2021_1706_MOESM2_ESM.docx]

Table 1. Search PubMed search string

| "Obesity/psychology"[Majr] OR "Obesity, Morbid/psychology"[Mesh] OR "Overweight/psychology"[Majr] OR "Body Mass Index"[Mesh] OR "Hyperphagia/psychology"[Mesh] OR "Food Addiction/psychology"[Mesh] OR obese OR “morbid obesity” OR overweight OR "over weight" OR overeat OR "over eat” OR "body mass index" OR BMI OR hyperphagia OR "food addiction" |
| --- |
| **AND** |
| "Narration"[Mesh] OR "Narrative Medicine"[Mesh] OR "Patient-Centered Care"[Mesh] OR "Interviews as Topic"[Mesh] OR "Personal Narratives as Topic"[Mesh] OR "Biographies as Topic"[Mesh] OR "Autobiographies as Topic"[Mesh] OR narration OR “narrative medicine” OR “patient-centered care” OR interview OR biography OR autobiography OR “personal narrative*” OR "lived experience" OR "patient narrative*" OR "patient story" |
